# Exploring extracellular vesicles as mediators of clinical disease and vehicles for viral therapeutics: Insights from the COVID-19 pandemic

**DOI:** 10.20517/evcna.2022.19

**Published:** 2022-07-19

**Authors:** Vaughn D. Craddock, Christine M. Cook, Navneet K. Dhillon

**Affiliations:** Division of Pulmonary, Critical Care and Sleep Medicine, Department of Internal Medicine, University of Kansas Medical Center, Kansas City, Kansas, KS 66160, USA.

**Keywords:** SARS-CoV-2, EVs, tissue factor, endothelial apoptosis

## Abstract

The COVID-19 pandemic has challenged researchers to rapidly understand the capabilities of the SARS-CoV-2 virus and investigate potential therapeutics for SARS-CoV-2 infection. COVID-19 has been associated with devastating lung and cardiac injury, profound inflammation, and a heightened coagulopathic state, which may, in part, be driven by cellular crosstalk facilitated by extracellular vesicles (EVs). In recent years, EVs have emerged as important biomarkers of disease, and while extracellular vesicles may contribute to the spread of COVID-19 infection from one cell to the next, they also may be engineered to play a protective or therapeutic role as decoys or “delivery drivers” for therapeutic agents. This review explores these roles and areas for future study.

## INTRODUCTION

**Figure 1.**
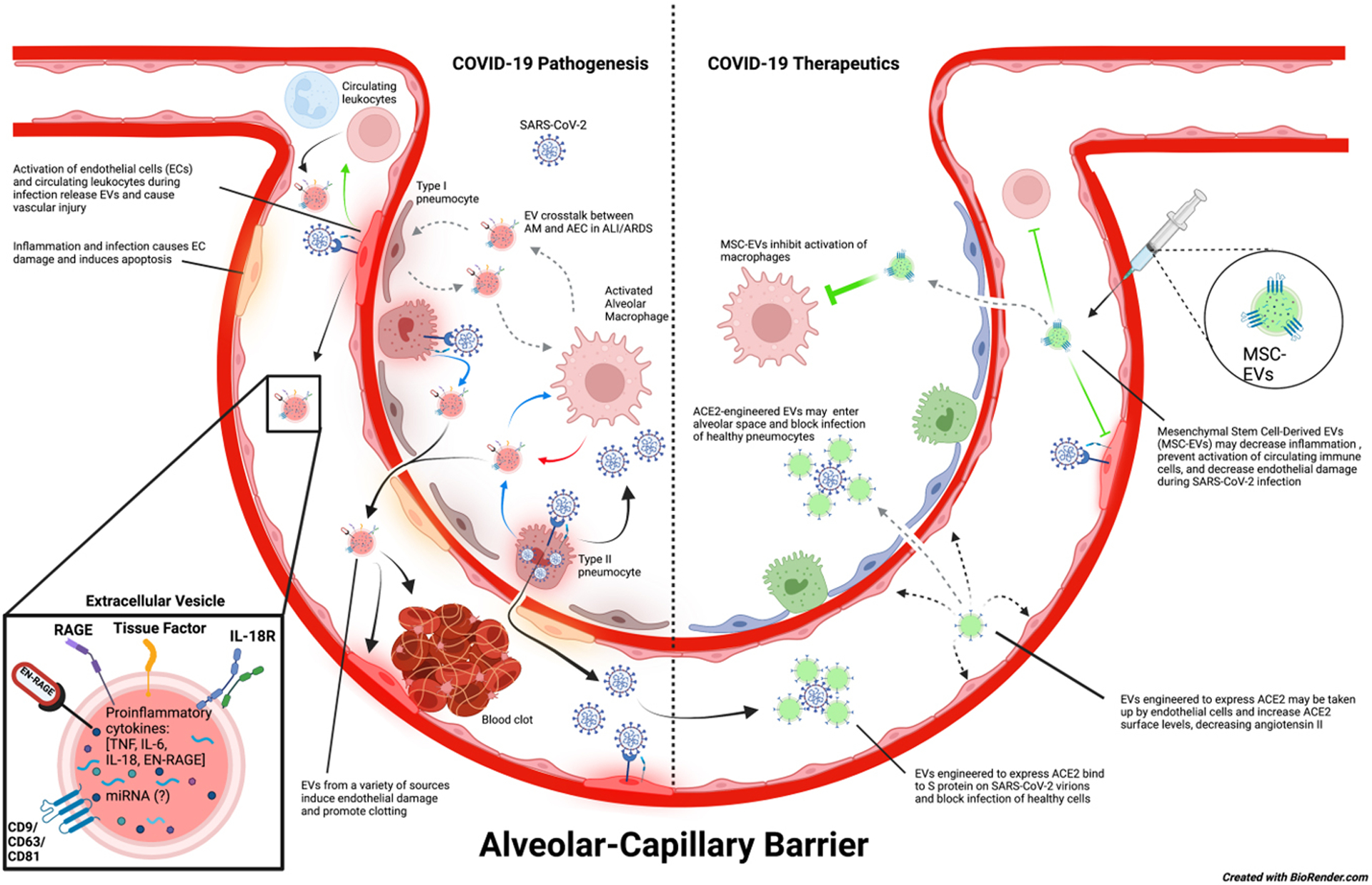
Potential role of EVs in COVID-19 disease pathogenesis and therapeutics. SARS-CoV-2 virions enter the host via the large airways and can travel into alveoli where they infect type II pneumocytes. Infection of type II pneumocytes by SARS-CoV-2 induces a proinflammatory response that activates alveolar macrophages and damages the alveolar epithelium. It is speculated that EV crosstalk between alveolar macrophages and alveolar epithelial cells could intensify inflammation and further enhance alveolar epithelial permeability. This may lead to increased permeability of the alveolar-capillary barrier, causing pulmonary edema and decreased functional capacity of the lungs, thereby allowing SARS-CoV-2 virions, EVs, and other inflammatory mediators to enter the bloodstream and progress to systemic infection. SARS-CoV-2 virions in the blood are suggested to infect endothelial cells and cause endothelial dysfunction, which ultimately leads to blood clots that can cause major thrombotic events as well as activation of circulating immune cells. On the contrary, engineered EVs could also be used as a therapeutic tool against COVID-19. EVs modified to express ACE2 may be injected into the bloodstream or nebulized and could potentially prevent SARS-CoV-2 from infecting healthy cells in either the bloodstream or alveolar space. These ACE2-expressing EVs may also be taken up by endothelial cells, increasing the amount of surface ACE2 expression on these cells, and maintaining the appropriate function of RAAS. In addition, injected MSC-EVs have anti-inflammatory properties that can promote tissue repair and prevent hyperinflammation during SARS-CoV-2 infection and ARDS. In COVID-19, they might be particularly useful for decreasing endothelial damage and preventing immune activation. (The copyright is retained by the authors).

The coronavirus disease 2019 (COVID-19) pandemic, caused by the severe acute respiratory syndrome coronavirus-2 (SARS-CoV-2), has led to the death of millions of people worldwide and has the potential to continue causing illness and mortality for the foreseeable future. SARS-CoV-2 infection most frequently causes fever with upper and lower respiratory symptoms, including rhinitis, sore throat, dyspnea, and cough. In some patients, COVID-19 can progress to a severe infection that requires hospitalization due to pneumonia, acute lung injury (ALI), or acute respiratory distress syndrome (ARDS)^[1–3]^. COVID-19 patients are also at risk for endothelial dysfunction and coagulopathies that increase the risk of adverse cardiovascular events^[4,5]^. Moreover, people with prior SARS-CoV-2 infection are now experiencing persistent COVID-19 symptoms following a recovery that can last many months after acute infection^[6,7]^. Recent studies also suggest an increased risk of developing pulmonary fibrosis, cardiovascular dysfunction, and other long-term complications following SARS-CoV-2 infection^[8–10]^. In this review, we highlight the role of extracellular vesicles (EVs) as drivers of COVID-19 clinical manifestations and the importance of EVs as potential biomarkers of disease prognosis according to published and ongoing research studies^[11]^. Finally, we will discuss the implications of EVs as novel therapeutics for patients with severe COVID-19 disease [Figure 1].

## ACUTE AND LONG-TERM MANIFESTATIONS OF COVID-19

SARS-CoV-2 establishes infection through endocytosis following the binding of the viral spike (S) protein to the ACE2 receptor on host cells^[12,13]^. In cases of severe COVID-19, SARS-CoV-2 infects type II pneumocytes, which express ACE2 in the lower respiratory tract^[13,14]^. Injury to type II pneumocytes upon infection results in decreased surfactant production by these cells, contributing to diffuse alveolar damage. In addition, the injury of type II pneumocytes is caused by an overwhelming immune response that leads to increased epithelial-capillary barrier permeability. This alveolar injury can lead to ARDS, a clinical hallmark of severe COVID-19 in hospitalized patients.

In conjunction with ARDS, patients with severe acute COVID-19 may also exhibit a hypercoagulable state and, therefore, be at increased risk for acute cardiovascular incidents. Hypercoagulability is stimulated by significant increases in proinflammatory cytokines TNF-α, IL-1β, and IL-6, which are all involved in signaling for the upregulation of coagulation factors^[15,16]^. These three cytokines are overexpressed in patients with severe COVID-19 and contribute to the infamous “cytokine storm”^[3]^. Simultaneously, SARS-CoV-2 infection has been suggested to induce endothelial damage either indirectly through immense inflammatory mechanisms or directly through the infection of endothelial cells^[17]^, which further increases the risk of blood clots during infection^[11,14,15]^. A recent study by Joffre *et al*. demonstrated that sera from patients with moderate and severe COVID-19 exhibit circulating biomarkers consistent with endothelial activation and dysfunction^[17]^. They further suggest from their findings that direct infection of endothelial cells by SARS-CoV-2 contributes to COVID-19-associated endotheliopathy. They confirmed by viral PCR that SARS-CoV-2 enters and replicates within human microvascular endothelial cells (HMVEC), which contributes to endothelial injury. How SARS-CoV-2 directly infects endothelial cells is still debated, with some saying that endothelial entry is mediated by ACE2 and others suggesting it is more likely to be through alternative receptors, such as neuropilin-1^[18–21].^ Several previous studies have reported that ACE2 is indeed found on the surface of vascular endothelial cells, and that SARS-CoV-2 dysregulates the renin-angiotensin-aldosterone system (RAAS) by inducing changes in endothelial ACE2 expression which contributes to endotheliopathy in COVID-19^[19,22–24]^. These findings suggest that SARS-CoV-2 entry into endothelial cells is at least partly responsible for the endothelial dysfunction in COVID-19 and contributes to hypercoagulability in these patients.

As the pandemic evolves, it has become clear that many COVID-19 patients experience symptoms for months following their acute infection, conditions referred to as Long COVID or Post-acute Sequelae of COVID-19 (PASC). A study by Carfì *et al*. assessed the prevalence of persistent COVID-19 symptoms in patients discharged from the Gemelli University Hospital (Foundation Policlinico Universitario Agostino Gemelli) in Rome, Italy, and found that, after approximately 60 days post recovery from acute infection, 87.4% of patients in their study still experienced at least one symptom of SARS-CoV-2 infection, and a majority of patients experienced three or more symptoms^[25]^. The most common long-term sequelae were fatigue and difficulty breathing^[25]^. Other studies have demonstrated that post-acute COVID patients exhibit decreased alveolar gas exchange based on pulmonary function testing and signs of interstitial lung damage and fibrosis on radiographic data for up to 12 months or longer post infection^[10,26]^. Long COVID patients also commonly report shortness of breath, fatigue, myalgias, sleep disturbances, malaise, mood changes, and chest pain 28 days or more after their acute infection^[27,28]^.

Of equal concern is the increased risk of cardiovascular disorders following SARS-CoV-2 infection. A recent large study that assessed patients within the U.S. Veterans Health Administration system found that COVID-19 was associated with an extra 23.98 incidents of major adverse cardiovascular events per 1000 people (i.e., stroke and heart attack) and 9.88 incidents of thromboembolic disorders per 1000 people, along with increased risks of many other cardiovascular abnormalities^[9]^. Although patients with severe COVID-19 are more likely to suffer from cardiovascular abnormalities post infection, even patients with mild cases are at increased risk of suffering from cardiovascular dysfunction^[9]^. It is anticipated that PASC will cause significant disease and mortality for years to come; therefore, it is vital to monitor post-acute COVID patients longitudinally to better understand the chronic manifestations of the disease. How the virus causes these persistent symptoms has yet to be fully understood, but it likely involves a variety of factors that may include the persistence of SARS-CoV-2 fragments in some tissues and associated inflammation for prolonged periods, reactivation of latent infections like Epstein-Barr virus, and/or worsening of preexisting health conditions^[29–31]^.

## EXTRACELLULAR VESICLES AS CONTRIBUTORS TO COVID-19 DISEASE PATHOGENESIS

### A brief description of EVs

EVs are small, lipid-enveloped nanoparticles ranging from 30–5000 nm in size found in plasma, serum, urine, and many other biophysiological fluids. They carry cargo, such as DNA fragments, RNAs (e.g., mRNA, miRNA, tRNA, or long noncoding RNAs), lipids, and proteins. These complex biomolecules are delivered between cells via EV trafficking and thus are important for intercellular communication^[32,33]^. EVs are divided into three classes that are differentiated by size, biogenesis, content, and function: apoptotic bodies, microvesicles, and exosomes^[34]^. Apoptotic bodies are 1–5 μm in diameter and are released from the plasma membrane by outward budding during apoptosis^[35]^. Although originally thought to be “junk” material released from dying cells, cargo from apoptotic bodies has been shown to stimulate progenitor cells and initiate tissue remodeling following cell injury^[36]^. Microvesicles (MVs) are large EVs that range in size from 100–1000 nm in diameter and are generated by budding from cell plasma membranes^[34]^. Exosomes are small EVs that range in size from 30–150 nm in diameter and are formed by cell sorting mechanisms, such as the endosomal sorting complex required for transport (ESCRT) pathways or ESCRT independent endosomal pathways^[34,35]^. Both MVs and exosomes have been studied for their involvement in intercellular communication and transporting cargo between cells. Extracellular vesicles are commonly studied for their use as biomarkers in metastatic cancer, sepsis, cardiovascular disease, COVID-19, and other conditions^[11,38–41]^.

Extracellular vesicles can be released by virus-infected cells, consequently transferring viral proteins, viral receptors, and proinflammatory cargo to recipient cells, thereby spreading infection and exacerbating tissue injury^[32,33,35]^. The EV lipid membrane offers protection for biomolecules against proteases, DNases and RNases, and other degradation catalysts that may exist in the bloodstream or other bodily fluids. Therefore, EVs can serve as vehicles for liquid biopsies by preserving cargo that may otherwise be degraded while circulating in the bloodstream as free biomolecules. EV cargo is derived directly from the cells generating and releasing them and therefore can offer a “snapshot” of the health and status of EV-generating cells, reflecting the pathogenesis of diseases in real time. Analysis of EV cargo can shed light on the body’s inflammatory and immune response to infection, provide evidence of endothelial dysfunction, and demonstrate features of coagulopathy. This has proven to be particularly helpful as we work to better understand the pathobiology of SARS-CoV-2 infection.

### EVs and COVID-19-associated inflammation and immune response

Since the start of the pandemic, multiple studies have demonstrated that a profound inflammatory response to the virus plays a major role in SARS-CoV-2 pathophysiology. The innate immune response to SARS-CoV-2 is robust, with many cytokines and chemokines serving as markers of disease severity. Patients with severe COVID-19 exhibit increased secretion of IL-2R, IL-6, and TNF-α^[42]^, and elevated circulating levels of IL-6 and TNF-α are independent predictors of mortality^[43]^. Recent findings from our lab demonstrate that both large and small EVs from the plasma of hypoxic patients with moderate-to-severe COVID-19 infection contain significantly elevated levels of members of the TNF superfamily and IL-6 family^[11]^. IL-6 can activate the protein kinase B (PKB)/Akt, mitogen-activated protein kinases (MAPK), and nuclear factor kappa-light-chain-enhancer of activated B cells (NF-κB) signaling pathways, all of which can promote inflammation and cellular crosstalk between immune and stromal cells^[44]^. We also observed a rise in the EV levels of RAGE and its ligand EN-RAGE (Extracellular Newly identified Receptor for Advanced Glycation End-products, a.k.a. S100A12) with the progression of COVID-19 infection, and significantly higher EV levels of EN-RAGE could distinguish patients with severe COVID-19 infection from those with moderate disease as well as deceased from critically ill patients who survived^[11]^. Advanced glycation end products (AGEs) are irreversible adducts formed from the glycation of proteins, lipids, and nucleic acids. AGEs interact with cell-surface receptor RAGE (receptor for AGE), causing an inflammatory cascade that generates reactive oxygen species (ROS) and tissue injury^[45]^.

EVs have previously been shown to contribute to ALI and ARDS in sepsis models^[2,46]^, and they may be involved in COVID-19-associated lung injury as well. EVs recovered from bronchoalveolar lavage fluid (BALF-EVs) during ARDS exhibit a complex interplay of communication between alveolar epithelial cells (AECs) and alveolar macrophages. In LPS-induced ARDS mouse models, BALF-EVs were enriched with miRNA that was shown to induce proinflammatory cytokines by AECs as well as alveolar macrophages (AMs) in cell culture experiments^[46–48]^. BALF-EVs containing increased amounts of miR-466g and miR-466m-5p were shown to activate the inflammasome and induce IL-1 in AMs^[48]^. Additionally, BALF-EVs enriched with miR-155 and miR-146a induced overexpression of TNF-α and IL-6 in AECs. These miRNAs also decreased the expression of the tight-junction protein, ZO-1, thereby increasing the permeability of the alveolar epithelial-capillary barrier^[46,47]^. Therefore, EVs have been shown to contribute to the increased alveolar vascular permeability, decreased alveolar ventilation and lung compliance, and the hyperinflammatory response that occurs in the lungs during ARDS. These disease processes broadly contribute to diffuse alveolar damage and pulmonary edema and, therefore, are highly applicable to COVID-19 ALI. Bronchoalveolar lavage (BAL) can be performed safely in patients with respiratory failure and lower respiratory tract infections^[49–51]^. Bronchoscopy and BAL extraction procedures have been limited during the pandemic to protect healthcare workers from exposure to SARS-CoV-2^[49]^. At the time of this writing, there is limited data on human BALF and BALF-EVs in COVID-19 pneumonia and ARDS. Nonetheless, we hypothesize that human BALF-EVs may be feasible biomarkers in COVID-19 patients on invasive mechanical ventilation, as previous studies demonstrate their role in ALI and ARDS. Since SARS-CoV-2 infection increases alveolar epithelial and vascular permeability, this might allow for BALF-EVs to “leak” into the bloodstream and further induce the production of proinflammatory cytokines that may lead to a more systemic immune response, thereby contributing to endothelial dysfunction and coagulopathy.

Apoptosis and necrosis of epithelial cells during ALI induced by SARS-CoV-2 is another important factor to consider in severe COVID-19. SARS-CoV-2 can induce cell death of lung epithelial cells by activating caspase-8, which can also contribute to immune activation through the induction of caspase-8-dependent expression of proinflammatory cytokines^[50–52]^. In addition, Fas Ligand (FasL) is an activator of caspase-8-mediated apoptosis in cells that express the death receptor Fas, the receptor for FasL, and is observed on the surface of EVs in chronic lung disease^[53–55]^. Cells expressing the death receptor Fas may include lung epithelial cells, virally infected cells, and T-lymphocytes which are all highly implicated in COVID-19 pathophysiology, especially during ALI/ARDS^[53]^. Interestingly, soluble FasL competes with membrane-associated FasL (mFasL) for binding to the death receptor Fas and, as a result, blocks cell death, highlighting that it is specifically mFasL that induces apoptosis^[53,56]^. Microvesicles and exosomes have been reported to express mFasL on their surfaces and induce apoptosis in T-lymphocytes[57,58]. Lymphopenia is a marker of COVID-19 severity and risk^[59]^, and one could speculate that EV-associated mFasL may contribute to T-cell death and serve as a potential therapeutic target in critically ill COVID-19 patients to maintain proper immune response.

Apoptotic bodies may also be important in viral-associated tissue damage. The role of apoptotic bodies is to safely compartmentalize dying cell debris which is then taken up by macrophages for digestion^[60]^. In theory, the packaging of debris from a dying cell into apoptotic bodies prevents activation of host immune cells, though some propose that apoptotic vesicles may elicit an immune response by delivering cytokines and damage-associated molecular patterns, or by activating immune cells, such as CD4+ T cells, through MHC-II molecules^[61–63]^. A very intriguing theory detailed later in this review is the potential capability of viruses to usurp apoptotic extracellular vesicles (ApoEVs) to promote their spread to healthy cells without calling attention to themselves by inducing an immune response.

### EVs and COVID-19-associated endothelial dysfunction

As mentioned above, in addition to epithelial injury, COVID-19 is associated with endothelial dysfunction, coagulopathy, and major adverse cardiovascular events like heart attack and stroke^[11,64]^. The endothelial damage and apoptosis could be due to direct infection of endothelial cells or indirect effects of inflammatory and thrombotic changes in circulation^[22,65]^. SARS-CoV-2 enters cells most commonly by binding to ACE2 receptors on respiratory epithelial cells and maybe endothelial cells as well. Infection subsequently leads to the downregulation of ACE2 receptors. Consequently, reduced ACE2 expression on endothelial cells would result in dysregulation of RAAS and could cause endothelial dysfunction due to inflammation and vasoconstriction induced by higher levels of angiotensin II^[23,24,66]^. Interestingly, EVs from patients with severe COVID-19 are observed to have lower levels of ACE2 compared to EVs from patients with a moderate disease^[11]^.

EVs appear to be heavily involved in COVID-19-associated endothelial activation and dysfunction. We demonstrated that both large and small EVs isolated from the plasma of patients with severe COVID-19 disease were significantly more capable of inducing apoptosis of pulmonary microvascular endothelial cells compared to EVs isolated from the plasma of healthy and asymptomatic groups^[11]^. In a recent study by Sur *et al*., the authors isolated exosomes from patients with mild-to-severe COVID-19 and observed that COVID-19 exosomes significantly induce the expression of NLRP3, caspase-1, and IL-1 mRNA in human endothelial cells^[67]^. Authors did not elucidate the cause of NLRP3 inflammasome activation by COVID-19 exosomes; however, it has been noted in conditions, such as diabetic retinopathy, that exosomes may carry damage-associated molecular patterns, such as high mobility group box-1 (HMGB-1)^[68–70]^, and induce the NLRP3 inflammasome via the canonical pathway^[71]^. Furthermore, EVs may traffic proinflammatory cytokines that activate the inflammasome via transactivation by NF-κB and contribute to endothelial dysfunction^[72]^.

EV-induced endothelial dysfunction most notably results in higher blood pressure due to reduced production of nitric oxide, a potent vasodilator, and increased clotting^[73]^. This causes increased resistance to flow, which can decrease perfusion to local organs and cause ischemic organ damage and increased systemic workload on the heart. We found that EV-associated ST2, a biomarker of myocardial dysfunction and organ damage, was positively correlated with increasing disease severity and length of hospitalization^[74]^.

Findings from Joffre *et al*.’s study, discussed earlier, might be of considerable interest in the EV field^[17]^. The authors point out that it is difficult to study the vascular endothelium in real time because of a lack of non-invasive procedures that can specifically and successfully help determine the health and status of endothelial cells during systemic infection, such as in COVID-19. In addition, the vascular endothelium is a vast monolayer of cells that serves as a barrier between blood and tissue and performs distinctive functions depending on the tissue site^[75,76]^. Therefore, assessing the function of the vascular endothelium systemically is very difficult with a tissue biopsy or ultrasound alone and requires one to first identify the site of endothelial damage. EVs may be the answer to this problem. EVs derived from endothelial cells (EC-EVs) express a variety of endothelial cell markers that can be captured with antibodies and isolated for further analysis^[76]^. Findings from EVs in COVID-19 pathogenesis^[11]^ propose some of the targets that could be used as biomarkers to assess endothelial function by analyzing EC-EVs in real time.

### EVs and COVID-19-associated coagulopathy

Endothelial damage from SARS-CoV-2 infection exposes connective tissue and induces the coagulation cascade following the binding of von Willebrand factor (vWF)^[77]^. Importantly, EVs and their cargo can directly contribute to the induction of the coagulation cascade in COVID-19 patients. Phosphatidylserine-exposing (PS+) EVs^[78]^ have been observed to be higher in COVID-19 patients with mild or moderate disease compared to those with severe disease^[79,80]^. Phosphatidylserine is a so-called “eat me” signal that interacts with phagocytes to stimulate phagocytosis of cells or vesicles^[81]^, but it is also a negatively charged molecule that facilitates activation of coagulation^[78]^. Recent studies demonstrated higher levels of tissue factor (TF) on EV surface in COVID-19 patients, and the levels of tissue factor-linked EVs (EV-TF) were found to be positively correlated with COVID-19 severity and mortality^[11,80,82]^. In addition, EV-linked vWF, urokinase plasminogen activator receptor (uPAR), and ADAMTS13 in COVID-19 patients correlated with the levels of thrombotic marker D-dimer, status of disease severity, and length of hospitalization.

Tissue factor is a primary initiator of coagulation in the blood and is associated with life-threatening thrombosis if overexpressed^[83]^. EVs have been shown to release or present TF and pro-coagulant phospholipids on their surface, promoting clot formation and accelerating fibrin polymerization^[84,85]^. EV-TFs in the blood are suggested to synergize with inflammation and endothelial injury, which consequently increases TF levels to overcome the thrombotic threshold^[86]^. EV-TF’s effect may be further potentiated by adhering to neutrophil extracellular traps (NETs) and concentrating at the site of clot formation. We observed higher levels of TF on EVs from COVID-19 patients who experienced stroke, venous thromboembolism, splenic infarct, or vision changes due to hypercoagulability^[11]^, consistent with previous findings showing an association of higher levels of EV-TF with venous thromboembolism^[87,88]^. Importantly, the positive correlation of EV-TF with disease severity and length of hospitalization^[11]^ was even stronger than the correlation of tissue injury marker lactate dehydrogenase, proinflammatory marker C-reactive protein, D-Dimer, and age with disease severity and length of hospitalization.

A study by Guervilly *et al*. similarly noted elevated EV-TF activity in COVID-19 patients and reported a positive correlation between elevated EV-TF activity and COVID-19 disease severity^[80]^. They also found that EV-TF activity was higher in patients with severe COVID-19 than in patients with septic shock, indicating that COVID-19 specifically influences the concentration of circulating EV-TF compared to other causes of infection^[80]^. Rosell *et al*. also reported elevated TF activity in EVs from plasma of COVID-19 patients, and further found that higher EV-TF activity correlated with a greater need for respiratory support (patients required > 5 L O_2_/min)^[82]^. They further found higher EV-TF activity in patients who ended up dying compared to those who survived. Although findings from our group also showed an increase in EV-TF activity in patients who died compared to those who survived, we did not find significant differences between the groups. This could be explained due to differences in the patient cohort. Our group compared severely diseased (WHO score ≥ 5) patients with deceased, whereas most of the patients in the Rosell *et al*. study had a moderate disease with a WHO score ≤ 5^[82]^. Nevertheless, these results suggest that EV-TF is a marker of disease severity and is associated with hypercoagulability, which could compound into severe COVID-19 disease.

Not only do COVID-19 EVs carry TF, but they also carry inflammatory molecules that induce the production of TF. Barberis *et al*. studied the exosomal proteome during COVID-19 and discovered that COVID-19 EVs traffic C-reactive protein (CRP) to distant cells and promote inflammation^[89]^. Patients with severe acute COVID-19 especially had elevated CRP in EVs. CRP is a highly soluble protein and a marker of COVID-19 severity^[90,91]^ and can induce endothelial and smooth muscle cells to express TF^[92]^. In addition, CRP causes endothelial dysfunction by inhibiting nitric oxide production, thereby preventing the normal function of vasodilatory responses that occur in response to increased pressures^[93]^. Therefore, it is unclear if EV-associated CRP is more biologically significant compared to soluble CRP in COVID-19 pathobiology, but it is a contributor to COVID-19 inflammation and endothelial dysfunction.

Barberis *et al*. also discovered that COVID-19 exosomes show a two-fold increase in kininogen-1, a precursor to bradykinin^[89]^. Des-Arg9 bradykinin (DABK), the activated form of bradykinin, is a substrate of ACE2 and is of particular importance during ALI and inflammation^[94,95]^. During an ALI, when ACE2 expression may be reduced on alveolar epithelial cells, especially during SARS-CoV-2 infection, bradykinin inactivation is impaired, and thus, DABK increases^[96]^. DABK is a known mediator of angioedema^[97]^ that significantly increases capillary permeability and induces the expression of several proinflammatory cytokines^[95,96]^. In fact, the kinin-kallikrein system is shown to be overactive in cases of severe COVID-19^[95,98,99]^. Moreover, exosomal kininogen-1 may be yet another biomarker of thrombosis risk in COVID-19 patients, because the kinin-kallikrein system is activated by Factor XII of the coagulation cascade^[97]^. Since the kinin-kallikrein system plays a role in the pathophysiology of ALI, we hypothesize that EVs carrying kininogen-1 may enter the bloodstream, contribute to systemic inflammation, and consequently increase the risk for thrombosis in COVID-19.

Finally, platelet-derived EVs (pEVs) are involved in coagulopathy in both infectious and noninfectious inflammatory conditions, including COVID-19^[100,101]^. Platelets are anuclear cells derived from megakaryocytes that serve a vital function in hemostasis^[102]^. The plasma-derived EVs are increased during acute SARS-CoV-2 infection^[100,103]^, but surprisingly, this increase is more pronounced in patients with non-severe COVID-19 compared to those in critical condition^[100]^. It is speculated that pEVs might be decreased in severe disease due to consumption because of blood hypercoagulability in COVID-19 patients. Therefore, pEVs appear to be partial contributors to coagulopathy in COVID-19 patients. In addition, pEVs may be released in response to endothelial activation and dysfunction or may themselves contribute to endothelial dysfunction and contribute to thromboembolic risk factors in COVID-19^[104]^.

### EVs and dissemination of SARS-CoV-2 infection

An emerging hypothesis that EVs aid in the spread and persistence of SARS-CoV-2 genetic material and proteins is of high interest. EVs and viruses have similar mechanisms of entry, budding, and biogenesis during infection. Previous studies have demonstrated that EVs can carry viral proteins and genetic material from infected cells to healthy cells during infections caused by CMV, HIV-1, or HSV-1^[105–108]^. Recent studies on spike-expressing cells and analysis of circulating EVs from a small set of COVD-19 patients suggest that EVs can incorporate S protein-derived peptides, but it is not yet clear if the spike peptide/protein gets incorporated on the surface or within the cargo of these particles^[109,110]^. S protein on EVs is shown to alter the immune response to SARS-CoV-2, which may modulate the immune response into one that is more severe^[109,110]^. A recent study by Barberis *et al*. reported the presence of low range copy numbers of SARS-CoV-2 RNA in the exosomes isolated from COVID-19 patient plasma, although in a very small number of patients^[89]^.

In another study by Yim *et al*., small EVs carrying endothelial marker CD31 were also observed to carry spike S1^[111]^. However, the authors did not find viral RNA in EVs by PCR detection^[111]^. Nonetheless, these data support the hypothesis that SARS-CoV-2 may be capable of infecting endothelial cells. Not only this, but the virus might be exploiting EV biogenesis by incorporating spike S1 into EVs which may serve as decoys that bind to neutralizing antibodies and protect SARS-CoV-2 virions from immune detection and destruction^[111]^. However, the authors went on to observe that patients with EVs carrying spike S1 also demonstrated high levels of antibodies against SARS-CoV-2 and less of an anti-inflammatory immune profile^[111]^. Therefore, it is unclear if it is SARS-CoV-2 exploiting EVs to protect itself or if the incorporation of spike S1 into EVs is an intentional attempt by infected cells to warn surrounding cells of viral infiltration. Further investigation is necessary to better understand this ambiguity surrounding the biological significance of spike S1-positive EVs.

In addition, EVs released by lung epithelial cells transduced with lentivirus encoding SARS-CoV-2 proteins have been shown to transfer the viral RNA to cardiomyocytes leading to upregulation of inflammatory gene expression in the recipient cells^[112]^. All these studies support the hypothesis that EVs carrying viral proteins or genetic material from SARS-CoV-2 contribute to the injury of healthy cells. This EV-facilitated crosstalk could intensify not only lung injury but also dysfunction of multiple other tissues and organs in patients with severe COVID-19^[113,114]^.

In a study with Syrian hamsters, S protein produced from a pseudovirus was shown to downregulate ACE2 via ACE2 receptor endocytosis by the S protein-carrying pseudovirus^[24]^. Additionally, S protein from the pseudovirus in hamsters increased oxidative stress through impaired mitochondrial function in endothelial cells, further exacerbating endothelial dysfunction^[24]^. Since the results of this study came from an attenuated pseudovirus, it is proposed that S protein alone can cause endothelial dysfunction and dysregulation of RAAS. All these findings suggest that S protein-positive EVs have the potential to contribute to endothelial dysfunction and disease severity in SARS-CoV-2 infections by dysregulating RAAS.

Frleta *et al*. found that HIV-1 induced cell death during acute infection and utilized apoptotic microparticles to inhibit dendritic cell activation by binding to CD44^[115]^. In ALI caused by COVID-19, there is a high degree of cell death within pulmonary tissue. Since COVID-19 decreases alveolar epithelial integrity and increases capillary permeability, SARS-CoV-2 might facilitate its own spread to systemic tissue through apoptotic vesicles, similar to HIV-1^[115]^. By utilizing host vesicles to conceal and traffic itself, SARS-CoV-2 may spread to other tissue and organs before the immune system can catch up. This idea is similar to the “Trojan Horse” mechanism in which viruses are thought to utilize EVs to disseminate their particles and genomes to other host cells[106]. Not only this, but pEVs have been shown to associate with viruses, including SARS-CoV-2^[100]^, and contribute to viral spread and proliferation^[79]^. Since platelets are constitutively present in the bloodstream and are hyperactive during SARS-CoV-2 infection^[100]^, it is reasonable to predict that platelets and the pEVs they release contribute to the systemic spread of the virus during acute infection. Future studies on this topic are warranted to fully elucidate the biological mechanisms of SARS-CoV-2 associations with pEVs.

A major challenge in EV research is the presence of other particles of the same size and shape, such as lipoproteins, bacteria-derived EVs, or virions and viral particles, that are often isolated with EVs. In EV research that involves viral infection, it can be difficult to isolate pure EVs from defective viral particles. Moreover, enveloped viruses, such as SARS-CoV-2, acquire a membrane derived from host cells, which is generated by budding from the plasma membrane and may include host cell markers^[116]^. To add to these challenges, viral particle biogenesis is shown to overlap with exosome biogenesis pathways, such as ESCRT, which means some enveloped viral particles may express common exosomal markers^[117]^. This is demonstrated in HIV, which recruits exosomal markers Alix, TSG101, and other ESCRT-1 proteins during viral budding, and makes it more difficult to truly ascertain if one is isolating pure EVs or a mix of EVs and defective viral particles^[117–119]^. Therefore, there is a possibility of co-isolation of virions along with EVs from bio-fluids using currently accepted methods of EV isolation, such as ultracentrifugation, precipitation with crowding reagents, size-exclusion chromatography, or affinity purification^[120]^. This is an inevitable challenge that must be acknowledged by researchers studying EV biology in the presence of viral infection. Nevertheless, viremia in COVID-19 patients is not as frequent, and if present, the reported copy numbers in plasma during acute infection are relatively low.

## EVS AS NOVEL THERAPEUTICS FOR COVID-19

One of the more exciting features of EVs is their potential usefulness for targeted therapeutics. EVs can be engineered to modulate the immune system for immunotherapies to deliver drugs or to inhibit tumor growth^[121–123]^. Now, EVs have been proposed for novel therapeutics for COVID-19. In one example, EVs were engineered to express the ACE2 receptor and serve as a decoy to prevent S protein from infecting healthy cells^[124]^. Cocozza *et al*. transduced 293FT cells with ACE2 and TMPRSS2, a host cell protease required for entry of SARS-CoV-2, using a lentivirus plasmid as the vector and isolated ACE2/TMPRSS2-expressing EVs released by these cells^[124]^. When cultured with healthy Caco-2 cells, they found that ACE2/TMPRSS2-expressing EVs decreased the rate of SARS-CoV-2 infection of healthy Caco-2 cells by 50%^[124]^. Another study proposed similar use of ACE2 EV decoys but using EVs derived from mesenchymal stem cells^[125]^. If administered to patients, these ACE2-loaded EVs could competitively bind S-protein of SARS-CoV-2 and prevent cell damage as well as dysregulation of RAAS by maintaining cell surface expression of ACE2. Therefore, ACE2-positive EVs may protect against ALI and endothelial dysfunction brought about by SARS-CoV-2 infection and high angiotensin II levels when ACE2 is subsequently decreased.

Another promising novel therapeutic is EVs derived from mesenchymal stem cells (MSC-EVs). Mesenchymal stem cells (MSCs) themselves have been proposed for clinical therapies in regenerative medicine, especially in mitigating lung injury during ARDS or other acute pulmonary exacerbations^[126,127]^. Therapies incorporating MSCs are attractive because MSCs are multipotential, naturally attracted to damaged tissue, and can “home” into tissues and aid in regeneration at directed sites^[126,128,129]^. However, concerns over their potential to cause iatrogenic cancerous tumors following administration may outweigh their potential therapeutic benefits^[127]^.

EVs derived from MSCs do offer several advantages over MSCs. First, they are less complex than MSCs, not self-proliferating, and, therefore, are easier to store and maintain. Second, because they are not proliferative, they do not pose a great risk of causing iatrogenic cancerous tumors. Thirdly, the inherent function of EVs is cell communication and so little effort needs to be allocated to modifying EVs collected from MSC-conditioned media following collection. Due to their nanoscale size, EVs can move freely even through small capillaries without obstructing flow, which allows for enhanced systemic communication. Finally, MSC-EVs do not express HLA-I and II and, therefore, can be used for allogeneic transfer without concerns of inducing an adverse immune reaction^[130]^.

Previously, MSC-EVs have been shown to have the potential for decreasing the severity of ALI. A study by Wei *et al*. demonstrated that MSC-EVs produced by human umbilical cord MSCs (hucMSCs) attenuated LPS-induced ALI in mice^[131]^. The group identified miR-377-3p, which was enriched in hucMSC-EVs, as an immunomodulator in BALF and inducer of autophagy, thereby reducing lung dysfunction and the severity of LPS-induced ALI^[131]^. Another study by Liu *et al*. showed that MSC-EVs decreased the expression of TLR4 and NF-κB as well as the production of proinflammatory cytokines TNF-α, IL-1β, and IL-6 in lung tissue in an intestinal-ischemia reperfusion lung injury model in rats^[132]^. These studies demonstrate the potential MSC-EVs have in attenuating the severity of ALI and preventing ARDS that can be induced by the “cytokine storm” in COVID-19 patients.

Sengupta *et al*. recently published a human clinical trial on ExoFlo^®^, a novel therapy involving the administration of bone marrow MSC-EVs (bmMSC-EVs) and reported positive outcomes in moderate-to-severe COVID-19 patients^[133]^. Following administration of a single 15 mL dose of bmMSC-EVs, ExoFlo^®^ was shown to increase patient survival and reduce the need for invasive oxygen support^[133]^. Additionally, because bmMSC-EVs have immunomodulatory properties, COVID-19 patients that were given ExoFlo^®^ also demonstrated a significant improvement in immune function and decreased levels of harmful acute inflammation^[133]^. Many other clinical trials involving the administration of anti-inflammatory and regenerative MSC-EVs are ongoing and can be found on ClinicalTrials.gov.

While MSC-EVs have strong potential for future therapeutics for many diseases, there are still several challenges to overcome. For one, the cumulative effects of MSC-EVs are far-reaching. MSC-EVs are most known for their immunomodulatory functions, but they are also shown to affect metabolism, tissue repair mechanisms, and angiogenesis^[134]^. Therefore, if administered to patients, the side effects experienced could be highly heterogenous unless MSC-EVs can be tailored to only have specific functions. Additionally, a major challenge in administering MSC-EVs to patients is the need for a high volume of conditioned media containing MSC-EVs to be given to a single patient, which is not conducive to currently approved methods of EV isolation and is also costly, though new methods, such as free-flow electrophoresis (FFE), are suggested may overcome this challenge^[135,136]^. Although promising, more research in this area is necessary to ensure that these therapies are safe and reproducible.

## CONCLUSION

In this review, we provide supporting evidence for EVs as biomarkers for COVID-19, EV involvement in acute and chronic COVID-19 pathologies, as well as the potential for engineered EVs and MSC-EVs as novel therapeutics in COVID-19 patients [Figure 1]. EVs are nanoparticles that clearly exhibit a diverse range of applications in science and medicine. Their inherent biological function as cell communicators allows for convenient assessments of disease pathologies via EV cargo, and their nanoscale size and non-proliferative nature make them safe and feasible for developing novel therapeutics. In addition, EVs carry a complex array of biomolecules, which can provide more information on organ and tissue function in one test compared to single biomarkers that are currently used to assess organ function. While more research is needed to develop logistical clinical diagnostics and therapeutics, EVs appear to be important mediators, promising biomarkers, and potential medicinal agents in COVID-19.
